# Use of CO_2_-derived variables in critically ill patients

**DOI:** 10.1186/s13613-025-01569-2

**Published:** 2025-09-25

**Authors:** Jihad Mallat, Mathieu Jozwiak, Nicolás Orozco, Olfa Hamzaoui, Xavier Monnet, Jean-Louis Teboul, Daniel De Backer, Gustavo A. Ospina-Tascón

**Affiliations:** 1 Critical Care Institute, Integrated Hospital Care Institute, Cleveland Clinic Abu Dhabi, Abu Dhabi, United Arab Emirates; 2 Cleveland Clinic Lerner College of Medicine of Case Western Reserve University, Cleveland, OH USA; 3 Faculty of Medicine, Republic of Srpska, University of Banja Luka, Banja Luka, Bosnia and Herzegovina; 4 Service de Médecine Intensive-Réanimation, CHU de Nice Hôpital L’Archet, 1, 151 Route Saint Antoine de Ginestière, Nice, 06200 France; 5 Université Côte d’Azur, UR2CA, Unité de Recherche Clinique Côte d’Azur, Nice, France; 6 Department of Intensive Care, Fundación Valle del Lili, Cali, Colombia; 7 Translational Research Laboratory in Critical Care Medicine (TransLab-CCM), Universidad Icesi, Cali, Colombia; 8 Unité de Médecine Intensive et Réanimation Polyvalente, CHU Reims, Reims, F-51100 France; 9 Université de Reims Champagne-Ardenne, Unité HERVI “Hémostase et Remodelage Vasculaire Post- Ischémie”, EA 3801, Reims, F-51100 France; 10 Service de Médecine Intensive-Réanimation, Hôpital de Bicêtre, DMU CORREVE, AP-HP, Université Paris-Saclay, Le Kremlin-Bicêtre, Paris, France; 11 Faculté de Médecine Paris-Saclay, Université Paris-Saclay, Le Kremlin-Bicêtre, Paris, France; 12 Department of Intensive Care, CHIREC Hospitals (Brussels and Braine l’Alleud-Waterloo), Braine-l’Alleud, Belgium; 13 Université Libre de Bruxelles, Brussels, Belgium

**Keywords:** Venous-to-arterial carbon dioxide tension difference, Carbon dioxide production, Oxygen supply dependency, Tissue hypoperfusion, Tissue hypoxia, Anaerobic metabolism, Oxygen consumption, Haldane effect, PCO_2_:CCO_2_ dissociation curve, venous oxygen saturation

## Abstract

A fundamental objective of hemodynamic resuscitation is to reverse tissue hypoperfusion and prevent progression to multiorgan failure and death. Conventional tools such as clinical examination, lactate levels, and central or mixed venous oxygen saturation (ScvO₂ and SvO₂, respectively) have intrinsic limitations. These drawbacks can be mitigated by incorporating additional markers of tissue hypoperfusion and altered tissue metabolism, such as carbon dioxide (CO₂)-derived variables, into other multimodal macro and micro hemodynamic monitoring. Specifically, the mixed or central venous-to-arterial difference in partial pressure of CO₂ (Pv-aCO₂ or Pcv-aCO₂) reflects the adequacy of blood flow to transport CO₂ from peripheral tissues to the lungs for its elimination. Consequently, Pv-aCO_2_ serves as a reliable marker for assessing the adequacy of blood flow relative to CO₂ production. Importantly, unlike SvO₂ and ScvO₂, Pv-aCO₂ remains informative even when oxygen extraction is impaired, as commonly occurs in septic conditions. However, Pv-aCO₂ and Pcv-aCO₂ in isolation are not direct markers of anaerobic metabolism, as these can also be influenced by oxygen consumption rates. Conversely, the ratio between Pv-aCO₂ (or Pcv-aCO₂) and the arteriovenous oxygen content difference may provide a more reliable indication of ongoing anaerobic metabolism. This review discusses the physiological foundations, prognostic significance, clinical implications, and potential applications of CO₂-derived parameters in patient management.

**Supplementary Information:**

The online version contains supplementary material available at 10.1186/s13613-025-01569-2.

## Background

Early recognition of tissue hypoperfusion and its reversion are key aspects to limiting the progression of shock and multiorgan dysfunction [1, 2]. For years, shock resuscitation has focused on increasing macro hemodynamics, aiming at reversing the imbalance between oxygen demand and supply to the tissues. Early goal-directed hemodynamic optimization targeting central venous oxygen saturation (ScvO_2_) was initially associated with a significant reduction of mortality in septic shock [3], but subsequent multicenter randomized trials failed to demonstrate some clinical benefit [4], since ScvO₂ is often normal or near normal in approximately two-thirds of septic patients on ICU admission [4, 5], achieving normal macro hemodynamics and/or global oxygen-derived parameters does not necessarily exclude the presence or persistence of tissue hypoxia.

Other markers, such as blood lactate concentration, have been demonstrated to be useful in detecting more severe cases and in guiding resuscitation during shock states [6–8]. Indeed, lactate-guided therapy significantly reduced hospital mortality in critically ill patients when adjusting for predefined risk factors [9]. In survivors, lactate levels almost invariably return to normal, and lactate kinetics have consistently been associated with morbidity and mortality across diverse clinical contexts [10]. However, blood lactate concentration may increase for many reasons unrelated to anaerobic metabolism [11, 12]. In addition, its dynamics is too slow for both an increase during shock progression and a decrease during the resuscitation phase [13]. Furthermore, blood lactate concentration relies on the balance between lactate generation and its reuptake by some tissues such as the liver and kidneys. Altogether, these limitations make lactate an imperfect marker of tissue anaerobic metabolism, tissue hypoperfusion, and a standalone guide for resuscitation.

In this context, using other resuscitation markers, such as carbon dioxide (CO_2_)-derived parameters, could provide additional information about macro and micro hemodynamics even in the presence of apparently corrected oxygen-derived variables and lactate levels [14–18]. Remarkably, CO_2_-derived parameters evolve more rapidly than resuscitation markers, such as blood lactate and microcirculatory blood flow distribution (evaluated by portable video-microscopy) [13], making CO_2_-derived parameters a potential tool for guiding resuscitation.

In this review, we will discuss the physiological principles, prognostic value, clinical significance, and potential clinical applications of CO_2_-derived parameters, including the mixed or central venous-to-arterial carbon dioxide difference (Pv-aCO_2_ or Pcv-aCO_2_) and the mixed or central venous-arterial carbon dioxide to arterial-venous oxygen difference ratios.

## Physiology of carbon dioxide

### Aerobic CO_2_ production

**Fig. 1:**
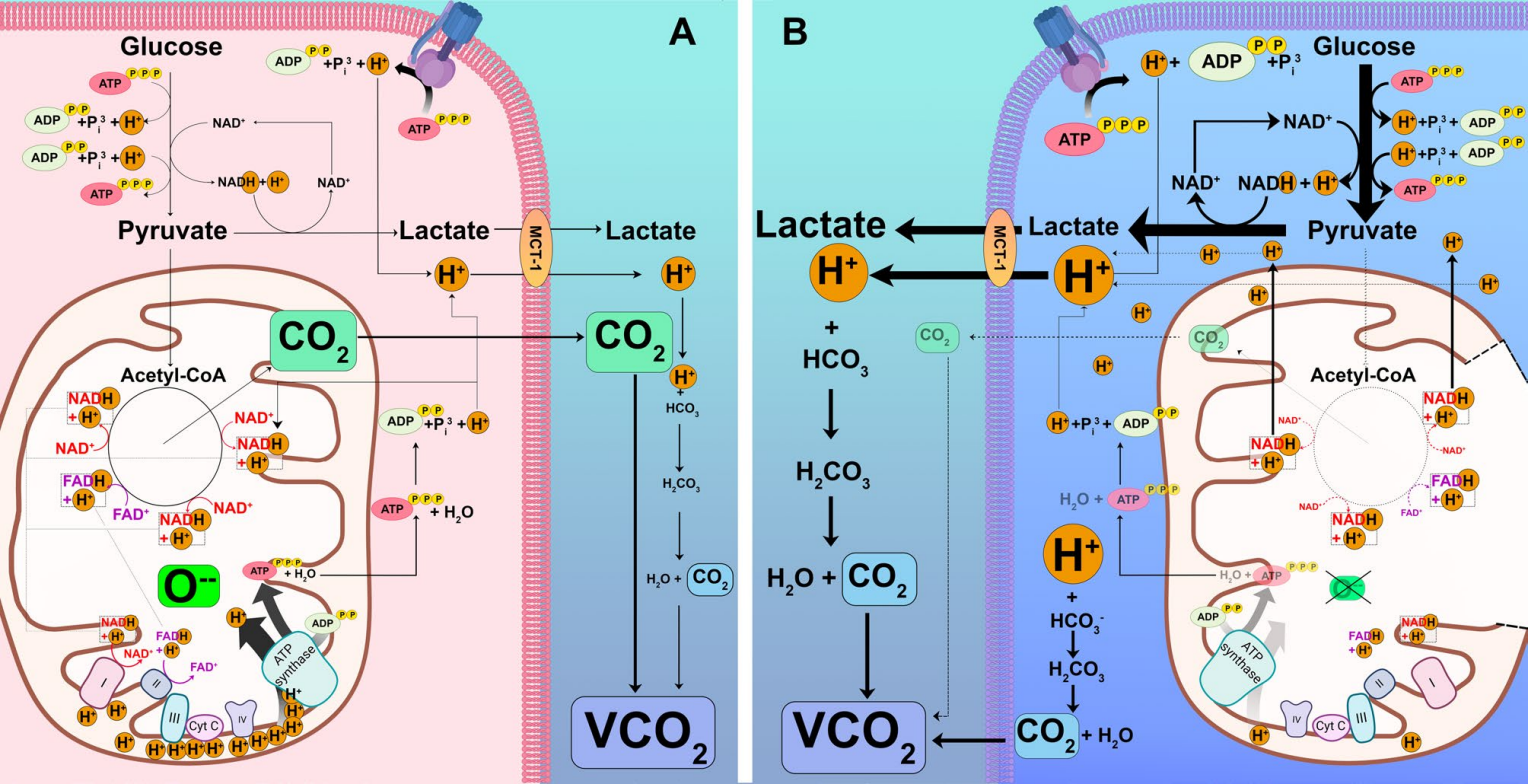
**A** Aerobic CO_**2**_ generation. Under aerobic conditions, carbon dioxide (CO_2_) is produced by the cells as the terminal metabolic product from substrate oxidation. ATP, adenosine triphosphate; ADP, adenosine diphosphate; H^+^, hydrogen ion; Complexes I, II, III, CytC, and IV, denote the components of the oxidative phosphorylation chain; MCT-1, monocarboxylate transporter-1; VCO_2_, CO_2_ production.** B** Non-aerobic CO_**2**_ generation (tissue hypoxia). During tissue hypoxia (right, blue cell), CO_2_ is generated by buffering the excess of H^+^ not recycled by other energy-generating processes (i.e., Krebs’s cycle). Such excess of non-reprocessed H^+^ are buffered by cytosolic and plasmatic bicarbonate (HCO_3_^−^), becoming H_2_CO_3_ to finally dissociate into CO_2_ and H_2_O. Thus, non-aerobic CO_2_ increase should theoretically contribute to the total VCO_2_

Under aerobic conditions, CO_2_ is produced by cells as the terminal metabolic product from substrate oxidation. Total CO_2_ production (VCO_2_) is directly related to global oxygen consumption (VO_2_) by the relation: VCO_2_ = RQ x VO_2_, where RQ represents the respiratory quotient, which reflects the ratio of moles of CO_2_ generated per mole of oxygen consumed. RQ may vary from 0.7 to 1 depending on the predominant energetic substrate consumed. Accordingly, VCO_2_ should increase when aerobic metabolism accelerates (i.e., increased tissue oxygen demand) or when carbohydrates are used as the predominant energetic substrate at a given VO_2_ [19] (Fig. 1A).

### Non-aerobic CO_2_ production

During tissue hypoxia, aerobic VCO_2_ decreases in proportion to the decline in VO_2_. Interestingly, when tissue hypoxia occurs, a portion of CO_2_ is non-aerobically generated through the buffering of protons (H^+^) by cytosolic and plasmatic bicarbonate (HCO_3_^−^). Thus, the excess of H^+^ is captured by HCO_3_^−^ to form H_2_CO_3_, which subsequently dissociates into CO_2_ and H_2_O (Fig. 1B). Nevertheless, the clinical demonstration of non-aerobic CO_2_ generation under tissue hypoxia is challenging as the total VCO_2_ also decreases [20, 21]. Additionally, the efferent venous blood flow may be sufficient to wash out the total CO_2_.

Remarkably, non-aerobic CO_2_ generation has been demonstrated in experimental metformin intoxication in which lactic acidosis was accompanied by a non-proportional decrease in VCO_2_ with respect to the concomitant VO_2_ fall (due to mitochondrial blockage by metformin), resulting in an increase in RQ, even under preserved systemic oxygen delivery (DO_2_) [22]. Of note, metformin intoxication induces mitochondrial dysfunction by inhibition of complex I, which is just another form of tissue hypoxia [23, 24]. Additionally, non-aerobic VCO_2_ can also result from anaerobic decarboxylation of substrates of intermediate metabolism such as α-ketoglutarate and oxaloacetate, although admittedly, its contribution to the total VCO_2_ is relatively small [25].

In summary, non-aerobic VCO_2_ might occur during tissue hypoxia, related or not to impairment of tissue perfusion (Fig. 1).

### Transport of CO_2_ content in blood

The transport of CO_2_ in the blood is closely determined by PCO_2_ variations, the intracellular and extracellular pH, and the surrounding partial pressure of oxygen (PO_2_) [26]. In general, blood carries both CO_2_ and its related compounds in five forms:

1. Dissolved CO_2_ (CO_2_)_DIS_, which is in equilibrium with the partial pressure of CO_2_ (PCO_2_) (as stated by Henry’s law) and represents ≈ 5% of the total CO_2_ content (CCO_2_). The actual concentration of (CO_2_)_DIS_ depends on the solubility coefficient of CO₂ in plasma, which in turn is influenced by temperature: higher temperatures reduce solubility, while lower temperatures increase it.
2. Carbonic acid (H_2_CO_3_), due to the reaction between CO_2_ and H_2_O (catalyzed almost instantaneously by carbonic anhydrase). Nevertheless, at physiological pH, H_2_CO_3_ instantly dissociates into H^+^ and HCO_3_^-^, whereby H_2_CO_3_ represents only 1/400th of the total CO_2_.
3. Bicarbonate (HCO_3_^−^), which is generated by the dissociation of H_2_CO_3_ into H^+^ and HCO_3_^−^ by a direct combination of CO_2_ and OH^−^ (also catalyzed by the carbonic anhydrase) and by a combination of carbonate (CO_3_^2−^) and H^+^. While H^+^ is buffered by hemoglobin, HCO_3_^−^ leaves the red blood cells through the HCO_3_^−^/chloride anion (Cl^−^) exchanger. Thus, HCO_3_^−^ increases in venous blood while Cl^−^ decreases. In arterial blood, bicarbonate (HCO₃⁻) accounts for approximately 90% of the total CO₂ content (i.e., ≈ 435 ml/L in whole blood). Although the absolute HCO₃⁻ concentration is slightly higher in venous blood due to increased CO₂ loading, the proportion of total CO₂ carried as HCO₃⁻ is slightly lower. This is because a greater fraction of CO₂ is bound to hemoglobin as carbamino compounds in venous blood, a shift facilitated by the Haldane effect (see below).
4. Carbonate (CO_3_^2−^), which is mainly generated from the dissociation of HCO_3_^−^ into CO_3_^2−^ + H^+^. Nevertheless, [CO_3_^2−^] is not quantitatively important for CO_2_ transport as it represents only ≈ 1/1000th of the total HCO_3_^−^ at pH 7.40.
5. Carbamino compounds, which represent CO_2_ linked to amino groups (R-NH_2_) of proteins, being the carbamino hemoglobin (Hb-NH-COO^-^), the most important carbamino compound, accounting for ≈ 5% of the total CCO_2_ in blood.

### The CO_2_ dissociation curve

Although the overall relationship between PCO₂ and total CCO₂ is curvilinear, it remains approximately linear within physiological ranges of PCO₂ and PO₂. Nevertheless, due to the Haldane effect, as hemoglobin gets saturated with O_2_, it will carry less CO_2_ as a carbamino compound since CO_2_ has a greater affinity for reduced than oxygenated hemoglobin [27]. Consequently, when blood enters the pulmonary capillaries and O_2_ is transferred to hemoglobin, the CO_2_-carrying capacity of hemoglobin decreases, facilitating the pulmonary excretion of CO_2_. The inverse occurs when blood becomes deoxygenated by releasing O_2_ to the tissues; then, hemoglobin gains the capacity to transport both extra aerobic and non-aerobic CO_2_. Simultaneously, the production of CO₂ and H⁺ by tissues promotes the release of O₂ from hemoglobin in the capillaries, a phenomenon known as the Bohr effect. Aerobic mitochondrial metabolism generates CO₂, increasing CCO₂ by ≈ 4 mL/dL from arterial to mixed venous blood (from 48 to 52 mL/dL), with a small PCO₂ rise (from 40 mmHg in the arterial blood to 46 mmHg in the mixed venous blood) due to the steep CO₂ dissociation curve. Without the Haldane effect, which enhances CO₂ carriage in deoxygenated hemoglobin, venous PCO_2_ would rise significantly more for similar CCO_2_ increases because less CO₂ could be stored as carbamino compounds and as bicarbonate.

**Fig. 2:**
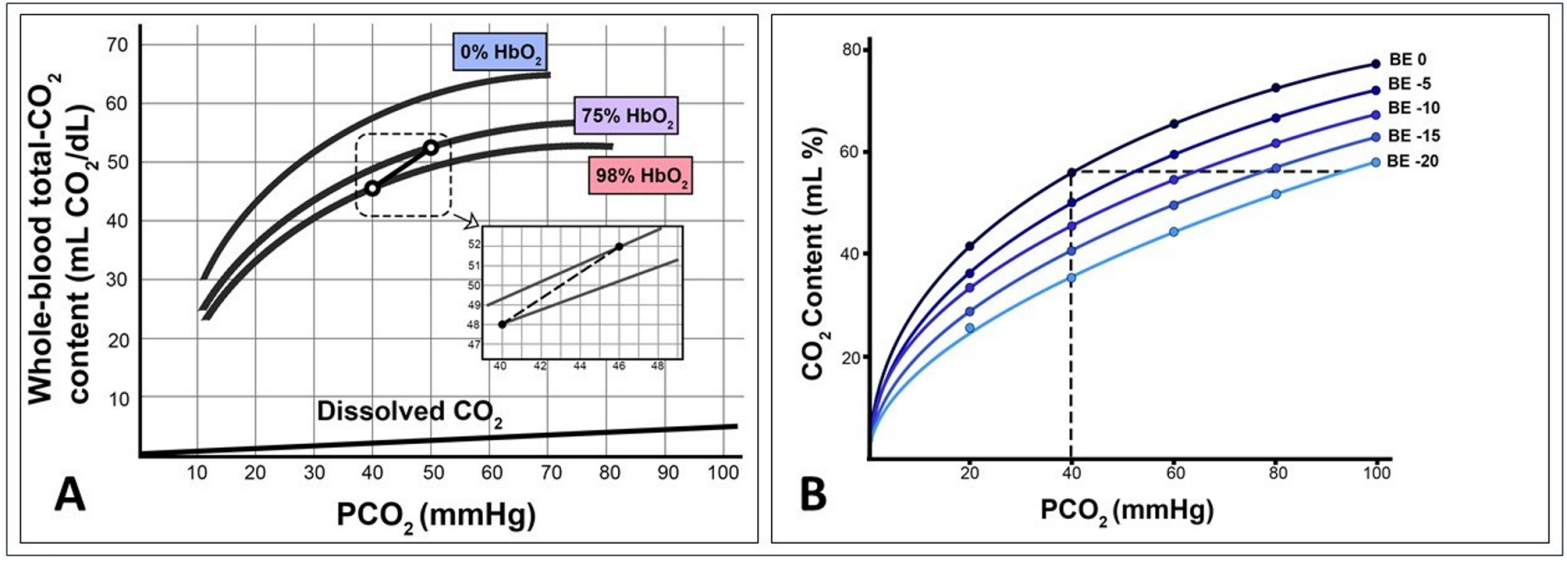
**A**: Effects of hemoglobin oxygen saturation on the relationship between CO_2_ pressures (PCO_2_) and contents (CCO_2_), i.e., Haldane effect. Curves depict the behavior of CCO_2_ at different hemoglobin oxygen saturations (including completely non-oxygenated hemoglobin - SO_2_:0% - under “ex vivo” conditions). Hemoglobin-O_2_ saturation affects the position of the CO_2_ dissociation curve. **B**: Effects of hydrogen ion accumulation on the PCO_2_:CCO_2_ relationship. Isopleths of base excess (BE) are described as PCO_2_ dramatically increasing for similar CCO_2_, as long as acidosis (hydrogen ion accumulation) also impairs

Remarkably, the relationship between PCO_2_ and CCO_2_ is also influenced by variations in H^+^ concentration, temperature, and hematocrit. Thus, blood CCO_2_ decreases as pH and HCO_3_^−^ also do, while an increase in temperature reduces the CO_2_ solubility, deviating the CCO_2_:PCO_2_ relationship downwards (Fig. 2). Similarly, an increase in hematocrit reduces the plasma space and increases pH, which ultimately reduces the CCO_2_ in blood.

### The venous-to-arterial carbon dioxide difference (Pv-aCO_2_)

Total venous CCO_2_ is determined by the mass of CO_2_ aerobically and non-aerobically generated in the tissues, and it is influenced by the metabolic rate and the RQ. Usually, CCO_2_ is higher on the venous side, thus creating a difference between the venous and arterial CCO_2_ (Cv-aCO_2_), which, under normal conditions of flow and metabolism, should be equivalent to the venous-to-arterial carbon dioxide pressure difference (Pv-aCO_2_) (the gradient of pressures exerted by the dissolved CO_2_ between the mixed venous-to-arterial blood). Nevertheless, arterial PCO_2_ might exceed venous PCO_2_ under hyperoxic apnea, a condition recreated during an apnea test to diagnose brain stem death [28] or during programmed intubation in the anesthesia setting [29], as a consequence of the Haldane effect. Interestingly, such “inversion” of the Pv-aCO_2_ is followed by symmetrical venous and arterial pH changes.

### Pv-aCO_2_ and its relationship with cardiac output

Pv-aCO_2_ depends on the total CO_2_ production, cardiac output, and the complex relationship between PCO_2_ and CCO_2_. The Fick equation states that CO_2_ excretion, i.e., the equivalent to VCO_2_ at a steady state, should equal the product of cardiac output and the venous-to-arterial CCO_2_ difference:

VCO_2_ = cardiac output x (CvCO_2_ – CaCO_2_).

As CCO_2_ and PCO_2_ depict a relatively linear relationship at usual physiological ranges (PCO_2_ = *k* x CCO_2_), PCO_2_ could be used as a surrogate of CCO_2_ [30, 31], and the Fick equation might be rewritten as:

Pv-aCO_2_ = $\:k$ x VCO_2_/cardiac output.

where $\:k$ is a pseudo-linear coefficient assumed to be constant under physiological conditions. Nevertheless, the factor $\:k$ may increase considerably under severe hypoxia and metabolic acidosis conditions, causing a significant shift in the curvilinear CCO_2_:PCO_2_ relationship (Fig. 2).

The modified Fick equation shows that Pv-aCO_2_ and cardiac output keep an inverse curvilinear relationship. Thus, under stable conditions of VO_2_ and VCO_2_, Pv-aCO_2_ will increase progressively in response to reductions in cardiac output due to venous CO_2_ stagnation, in which a delayed transit time of red blood cells in the tissues leads to a greater addition of CO_2_ per unit of blood flowing through efferent microvessels.

Early observations during cardiac arrest in both animal models and humans revealed a close relationship between no blood flow and venous CO_2_ accumulation [31, 32]. Similar results were shown in experimental models of hemorrhage, hypovolemia, and obstructive shock, thus highlighting the importance of blood flow stagnation on venous CO_2_ accumulation, which in turn increases Pv-aCO_2_ [20, 21, 33, 34].

### Can the PCO_2_ gaps be used as a marker of tissue hypoxia?

The increase in Pv-aCO_2_ was initially interpreted as a marker of tissue hypoxia, as critical DO₂ values seemed to coincide with the point at which venous PCO_2_ began to rise in experimental studies [20, 33, 35]. However, these experiments could not distinguish between tissue hypoperfusion and tissue hypoxia. To address this, Vallet et al. [36] compared the effects of hypoxic hypoxia and ischemic hypoxia on the VO_2_:DO_2_ relationship and regional Pv-aCO_2_ in a vascular-isolated canine hindlimb model perfused via a roller pump–membrane oxygenator circuit. To examine the isolated effects of hypoxia or flow, decreases in DO_2_ and VO_2_ were induced by progressive reductions of the inspired O_2_ fraction (FiO_2_) but preserving flow (hypoxic hypoxia) and by gradual reductions of the roller pump velocity but maintaining FiO_2_ (ischemic hypoxia). Even though both groups experienced similar decreases in DO_2_ and VO_2_, regional hind limb Pv-aCO_2_ remained constant during hypoxic hypoxia, while it increased more than two-fold during ischemic hypoxia, suggesting that blood flow is the major determinant of Pv-aCO_2_. Similarly, Nevière et al. [37] showed significant increases in gut mucosal-to-arterial PCO_2_ difference (Pmtis-aCO_2_) during ischemic hypoxia, while it remained almost constant over a wide range of DO_2_ values during hypoxic hypoxia. Interestingly, Pmtis-aCO_2_ increased slightly during extreme hypoxic hypoxia, which could indicate some local non-aerobic CO_2_ generation. Similar results were observed in experimental hemodilution, in which no increase in Pmtis-aCO_2_ was detected under conditions of VO_2_:DO_2_ dependency due to reduced hemoglobin concentration unless there was a concomitant reduction in cardiac output [38].

Both experimental [36, 37] and clinical [39–41] data suggest that in tissue hypoxia, Pv-aCO_2_ can be high or normal, depending on cardiac output. Tissue hypoxia occurring during low flow states increases Pv-aCO_2_ due to CO_2_ venous stagnation. However, in cases of tissue hypoxia with preserved or increased blood flow, Pv-aCO_2_ may be normal or even reduced, as the flow in venous efferent vessels could be sufficient to wash out the CO_2_ generated by the tissues, as occurred in hypotensive patients with fulminant hepatic failure and concomitant tissue hypoxia [41]. Consequently, a normal Pv-aCO_2_ does not rule out tissue hypoxia or dysoxia.

In summary, Pv-aCO_2_ is a useful surrogate of the adequacy of cardiac output and tissue perfusion for a given level of CO_2_ production. However, the correlation between variations in cardiac output and Pv-aCO_2_ during septic shock is weak [15, 39, 40, 42], suggesting that factors beyond cardiac output also influence Pv-aCO_2_ [18].

### Tissue-to-arterial CO_2_ gaps, Pv-aCO_2,_ and microcirculatory blood flow alterations

Microcirculatory dysfunction in septic shock is a generalized phenomenon essentially characterized by decreased functional capillary density (FCD) and increased heterogeneity of blood flow, which implies the presence of areas with well-perfused vessels in close vicinity to non-perfused capillaries [43, 44]. Créteur et al. [45] showed a good correlation among sublingual microcirculatory blood flow distribution evaluated by video microscopy, the sublingual-to-arterial PCO_2_ difference (Psl-aCO_2_), and gastric mucosal-to-arterial CO_2_ (Pgtis-aCO_2_). Similarly, Nevière et al. [46] reported that variations in gastric microvascular blood flow were tracked by variations in gastric mucosal-to-arterial PCO_2_ differences (Pgtis-aCO_2_), suggesting that microvascular blood flow may play an important role in gastric-tissue CO_2_ accumulation.

Some investigators have suggested a close link between microcirculatory blood flow derangements and Pv-aCO_2_ during the early stages of septic shock [18, 47]. Increases in Pv-aCO_2_ were paralleled by a gradual decrease in sublingual functional capillary density and increased microvascular flow heterogeneity, independently of cardiac output variations [18]. Likewise, Pv-aCO_2_ was related to microcirculatory alterations and reduced tissue oxygenation, although this relationship was dependent on cardiac output [47]. However, in that study, microcirculation was evaluated using near-infrared spectroscopy, a technique that does not directly measure microcirculatory blood flow [47].

During severe tissue hypoxia, total VCO_2_ decreases even when non-aerobic CO_2_ generation is present [20, 21, 33]. Nevertheless, both low cardiac output and potentially increased heterogeneity of microvascular blood flow respond, to some extent, to the increases in venous CCO_2_ [18].

### What is the difference between tissue PCO_2_ and venous PCO_2_?

Most of the CO_2_ is produced by the tissues, in aerobic as well as in hypoxic conditions. CO_2_ easily diffuses to capillaries and venules so that the gradient between tissue PCO_2_ and PvCO_2_ is usually very low (2–4 mmHg). When tissue hypoxia occurs, at the point of critical DO_2_, tissue PCO_2_ sharply increases. Interestingly, CO_2_ can diffuse longer distances than O_2,_ so that it can still reach the venous side in very low flow conditions, even when some areas are almost not perfused. Nevertheless, the gradient between arterial PCO_2_ and tissue PCO_2_ would be much higher than Pv-aCO_2_. In an experimental tamponade, Schlichtig et al. have shown that tissue PCO_2_ in the gastric mucosa reached levels between 90 and 210 mmHg while portal vein PCO_2_ reached “only” 80 mmHg [35]. However, a locally increased PCO_2_ in the gastric mucosa might not reflect hypoperfusion in the entire regional circulation. In an experimental model of sepsis, with preserved cardiac output but associated with splanchnic hypoperfusion, Ospina et al. reported that the ileal tissue-arterial PCO_2_ gradient increased more than Pv-aCO_2_ in the mesenteric vein [48].

### Other factors influencing the PCO_2_ gap

#### Factors related to the PCO_2_:CCO_2_ relationship

Pv-aCO_2_ or central Pv-aCO_2_ (Pcv-aCO_2_), calculated as the difference between central venous PCO2 sampled from a central vein catheter and arterial PCO_2_, may be influenced by factors beyond blood flow and anaerobic metabolism, as they affect the PCO_2_:CCO_2_ relationship. While this relationship remains quasi-linear within the physiological PCO_2_ range [49], it becomes curvilinear when altered by certain conditions [26]. Key influencing factors include oxygen saturation (Haldane effect) [50, 51], the degree of metabolic acidosis [52], and hematocrit levels [51].

##### Haldane effect

Low local hemoglobin oxygen saturation increases CCO_2_ for a given PCO_2_ as deoxygenated hemoglobin binds more CO₂ due to the Haldane effect (Fig. 2A) [53, 54]. In conditions of very low SvO_2_ (< 30%), Jakob et al. [55] found that changes in gastric mucosal Pv-aCO_2_ may not always reflect changes in CCO_2_ differences or blood flow, potentially due to the Haldane effect. However, gastric mucosal oxygen saturation was not directly measured but rather estimated based on gastric mucosal oxygen extraction, which may have influenced these results. In an experimental study using a vascularly isolated, innervated, and perfused dog hindlimb, Mallat and Vallet [56] observed that the Haldane effect did not significantly influence the PCO_2_:CCO_2_ relationship, even when regional SvO_2_ dropped well below 30%. Similarly, in healthy individuals during intense exercise, Sun et al. [57] reported that variations in SvO_2_ had only a minor impact on the PCO_2_:CCO_2_ relationship. Additionally, in septic shock patients, Mesquida et al. [58] found that SvO_2_ (Haldane effect) had a minimal impact on the PCO_2_:CCO_2_ relationship, further suggesting that its clinical significance may be limited.

##### Metabolic acidosis effect

Hydrogen ions accumulation can shift the PCO_2_:CCO_2_ relationship so that PCO_2_ is higher for a given CCO_2_ (Fig. 2B). In an experimental study using a perfused dog hindlimb model [56], metabolic acidosis significantly impacted the PCO_2_:CCO_2_ relationship. When venous pH changes were ignored, the relationship remained nearly linear. However, when pH variations were accounted for, CCO_2_ was no longer linearly related to PCO_2_. As metabolic acid accumulated from hypoxic cells, PCO_2_ and CCO_2_ changed in opposite directions due to decreased plasma and red blood cell CCO_2_ and bicarbonate levels. Similarly, during heavy exercise in healthy individuals, Sun et al. [57] found that once the lactic acidosis threshold was reached, pH changes disrupted the linearity of the PCO_2_:CCO_2_ relationship, with CCO_2_ and PCO_2_ sometimes moving in opposite directions. In patients with septic shock, Mesquida et al. [58] identified metabolic acidosis (pH) as the primary predictor of the discrepancy between mixed Pv-aCO_2_/Ca-vO_2_ and Cv-aCO_2_/Ca-vO_2_. Thus, in situations with moderate/severe metabolic acidosis, an elevated Pv-aCO_2_ might not reflect only low or inadequate cardiac output but could also be ascribed to modifications of the CO_2_–hemoglobin dissociation curve, suggesting the reliability of Pv-aCO_2_ and its combination with O_2_-derived variables as markers of tissue perfusion and cell metabolic dysfunction during shock.

##### Hemoglobin effect

The impact of hemoglobin on the PCO_2_:CCO_2_ relationship has been less extensively studied. In surgical patients with sepsis and varying degrees of illness, Chiarla et al. [51] observed that a decrease in hemoglobin levels increased Pv-aCO_2_ for a given CCO_2_. However, this effect was minimal under normal physiological conditions and became significant only in extreme scenarios. In such cases, a combination of low hemoglobin concentration, low-flow states, acidosis, and hypercapnia may synergistically disrupt the physiological balance, amplifying the impact on the PCO_2_:CCO_2_ relationship, leading to a disproportionate rise in Pv-aCO₂ relative to CCO₂.

#### Factors related to an acute change in PaCO_2_

Under spontaneous breathing conditions, hyperventilation triggered by reduced blood flow may decrease PaCO₂, thus preventing the rise in PvCO₂ that typically results from CO₂ stagnation [59]. This observation highlights the advantage of evaluating the PCO₂ gap instead of relying solely on PvCO₂ measurements, especially when patients are breathing spontaneously [60].

However, acute changes in PaCO_2_ due to ventilator adjustments may influence the PCO₂ gap. In a small study of 10 mechanically ventilated postoperative patients, Morel et al. [61] found that acute hyperventilation significantly increased Pcv-aCO_2_ without affecting cardiac output. Similarly, in patients with septic shock, Mallat et al. [62] demonstrated that acute hyperventilation increased Pcv-aCO_2_ independently of cardiac output. This rise was attributed to increased VO_2_ triggered by acute respiratory alkalosis. These findings regarding the effects of hyperventilation on the Pcv-aCO_2_ were later confirmed by Guo et al. [63]. Clinicians should be mindful of the impact of alveolar hyperventilation on PCO₂ gap when interpreting its values at the bedside, as it may not always reflect the perfusion status alone.

### The venous-arterial PCO_2_ and CO_2_ content to arterial-venous O_2_ content ratio (Cv-aCO_2_/Ca-vO_2_ and Pv-aCO_2_/Ca-vO_2_ ratios)

#### Physiological rationale

According to the Fick equation, VO_2_ and VCO_2_ are directly proportional to cardiac output and their respective arterial-to-venous and venous-to-arterial content differences. Under aerobic steady-state conditions, VCO_2_ approaches VO_2_, whereby the mixed venous-to-arterial CCO_2_ (Cv-aCO_2_) approximates to the arterial-to-mixed-venous O_2_ content difference (Ca-vO_2_). VCO_2_ should not exceed O_2_ availability, and consequently, the VCO_2_/VO_2_ ratio should not be > 1.0 under normal metabolic steady-state conditions. In these terms, the Cv-aCO_2_/Ca-vO_2_ ratio could represent some approximation of the VCO_2_/VO_2_ ratio or RQ, and it should be independent of blood flow variations as the cardiac output is present in both the numerator and denominator components [16, 52].

Experimental blockade of mitochondrial O_2_ utilization leads to non-symmetrical reductions in VCO_2_ and VO_2,_ with the subsequent increase in the RQ [22]. Other experimental shock models induced by central hypovolemia also showed that VCO_2_ decreased less than the VO_2_ decrease, with the subsequent increase in the VCO_2_/VO_2_ ratio [21]. An asymmetric fall in VCO_2_ and VO_2_ could be explained by increased non-aerobic VCO_2_ as a result of buffering of excess H^+^ due to severely limited tissue O_2_ availability (tissue hypoxia), or alternatively, because of mitochondrial dysfunction. Interestingly, the VCO_2_/VO_2_ ratio returns to normal after shock resolution, such as seen in the splanchnic regional Cv-aCO_2_/Ca-vO_2_ ratio after resuscitation in experimental peritonitis models [48] and endotoxemic shock [64]. Other authors have also observed simultaneous increases in the Cv-aCO_2_/Ca-vO_2_ ratio, RQ, and lactate levels during circulatory failure in mechanically ventilated patients [65, 66], thus reinforcing the idea of a link between non-aerobic VCO_2_ and increased Cv-aCO_2_/Ca-vO_2_ ratio. Contrastingly, a sub-analysis of an experimental model of progressive hemorrhage suggested that the Pv-aCO_2_/Ca-vO_2_ ratio is a poor surrogate of anaerobic metabolism during marked hemodilution [50]. Nevertheless, at extremely low hemoglobin levels (~ 2 g/dL, rarely seen in clinical practice), inherent measurement errors may be amplified in the total Pv-aCO_2_/Ca-vO_2_ ratio. In such conditions, indirect calorimetry may also be inappropriate due to the highly variable time required for equilibration among different CO_2_ stores (CO_2_ dissolved in plasma, bicarbonate, and tissue CO_2_) during the induction and resuscitation of shock. Furthermore, PCO_2_ can rise sharply at very low hematocrit levels, with minimal changes in total CCO_2_, because reduced hemoglobin impairs CO_2_ buffering and binding capacity [51].

In conclusion, the Cv-aCO_2_/Ca-vO_2_ ratio and its potential equivalent, the Pv-aCO_2_/Ca-vO_2_ ratio, could identify the presence of non-aerobic VCO_2,_ whether mediated by tissue hypoxia (limited O_2_ availability) or mitochondrial dysfunction (with preserved O_2_ availability) (Fig. 1).

#### Clinical relevance of PCO_2_ gaps

##### Septic shock

High ScvO_2_ does not exclude the presence of tissue hypoperfusion and hypoxia in cases of impaired oxygen extraction, which can occur in septic shock [67, 68]. Due to its high solubility—approximately 20 times that of oxygen—CO_2_ readily diffuses from ischemic tissues into efferent veins, making it a highly sensitive marker of hypoperfusion. As a result, in conditions where oxygen diffusion is impaired due to shunted or obstructed capillaries, the PCO_2_ gap can help to reveal underlying hypoperfusion.

In a prospective study that included 64 patients with septic shock, non-survivors had a significantly higher Pv-aCO_2_ than survivors (5.9 ± 3.4 mm Hg vs. 4.4 ± 2.3 mm Hg, *p* < 0.05), related to a higher PvCO_2_ level. Furthermore, patients with an increased Pv-aCO_2_ (≥ 6 mmHg) were more likely to die (73.3% vs. 32.6%, *p* < 0.01) [39]. Also, Ospina-Tascón et al. [15] demonstrated that persistently elevated Pv-aCO_2_ (≥ 6 mmHg) after the first six hours of resuscitation in patients with septic shock was associated with more severe multiple organ failure and higher mortality rates. However, measuring mixed Pv-aCO_2_ requires a pulmonary artery catheter, which is more rarely used in current clinical practice [69]. Since most patients with septic shock have a central venous catheter in place, central Pv-aCO_2_ (Pcv-aCO_2_) offers a more practical alternative with comparable clinical utility. Interestingly, a strong agreement between Pv-aCO_2_ and Pcv-aCO_2_, was reported in critically ill patients [70] and in patients with severe sepsis or septic shock [42]. However, in septic patients, the 95% limits of agreement were wide, suggesting that while both mixed and central venous PCO_2_ can be used to calculate the PCO_2_ gaps, they should not be used interchangeably during treatment.

While reduced oxygen extraction and increased tissue oxygen debt may mask hypoxia, CO_2_ continues to diffuse into the efferent veins, exposing the hypoperfusion state to clinicians when Pcv-aCO_2_ is assessed [71]. Vallée et al. [14] tested the hypothesis that Pcv-aCO_2_ could serve as a global indicator of tissue hypoperfusion in resuscitated septic shock patients with ScvO_2_ > 70%. They found that despite normalizing the DO_2_/VO_2_ ratio, patients with persistent tissue hypoperfusion, indicated by blood lactate concentrations > 2 mmol/L, also exhibited elevated Pcv-aCO_2_ (> 6 mmHg). Moreover, patients with lower Pcv-aO_2_ values showed larger reductions in blood lactate concentrations, higher cardiac output values, and significantly larger decreases in SOFA scores than those with persistently high Pcv-aCO_2_. In a prospective study of 80 patients, Mallat et al. assessed the clinical value of measuring Pcv-aCO_2_ during the early resuscitation phase of septic shock [72]. The findings revealed that patients who achieved a normal Pcv-aCO_2_ (≤ 6 mmHg) within six hours of resuscitation experienced greater decreases in blood lactate concentrations and SOFA scores than those who did not [72]. Notably, patients who met both Pcv-aCO_2_ ≤ 6 mmHg and ScvO_2_ > 70% targets within the first six hours had the most significant reductions in blood lactate concentrations, which emerged as an independent prognostic factor for ICU mortality [72].

Supporting these findings, Du et al. [73] conducted a retrospective study showing that the simultaneous achievement of ScvO_2_ normalization and central Pcv-aCO_2_ ≤ 6 mmHg was a stronger predictor of favorable outcomes after septic shock resuscitation than ScvO_2_ alone, as blood lactate was cleared more effectively in patients meeting both targets. Wang et al. [74] reported in a prospective study of 152 septic shock patients that Pcv-aO_2_ was independently associated with 28-day mortality in a multivariable regression analysis. Other prospective studies found a significant association between Pcv-aCO_2_ and mortality in patients with sepsis or septic shock [75–78].

An important question is whether PCO_2_ gap can serve as a target for resuscitation. In a recent randomized controlled trial (RCT) that included 179 patients with acute circulatory failure who were assigned to receive either a CO_2_-O_2_-derived algorithm-based treatment or standard of care clinical practice for hemodynamic resuscitation, there was no significant difference between the two groups regarding the primary outcome, which was lactate clearance > 10% at two hours. Additionally, the secondary outcomes (SOFA score, ICU and hospital length of stay, and 28-day mortality) did not show significant differences between the two groups [79]. Further trials are needed to assess the effectiveness of targeting Pcv-aCO_2_ as the end-goal of hemodynamic resuscitation in septic shock patients. Importantly, one should recall that PCO_2_ gaps, as lactate, should not be looked at in isolation [80]. PCO_2_ gaps, lactate, capillary refill time, and other indices of tissue hypoperfusion should be integrated, and attempts to continue resuscitation may be paused when the majority of indices are normalized.

##### Major non-cardiac surgery

Robin et al. [81] conducted a prospective study including 115 high-risk non-cardiac surgery patients, primarily undergoing abdominal surgery. They reported that Pcv-aCO_2_ measured upon ICU admission was significantly higher in patients who developed postoperative complications than those who did not (8.7 ± 2.8 mmHg vs. 5.1 ± 2.6 mmHg, *p* = 0.001). Additionally, Pcv-aCO_2_ on ICU admission predicted postoperative complications with an AUROC of 0.86, with an optimal cutoff value of 5.8 mmHg. Notably, Pcv-aO_2_ was a superior predictor of postoperative complications compared to arterial lactate concentrations. A higher than normal Pcv-aCO_2_ (≥ 6 mmHg) was associated with increased organ failure, prolonged mechanical ventilation, and extended hospital stay. Several observational studies have identified an association between Pcv-aCO_2_ and outcomes in high-risk non-cardiac surgical patients [82–84]. However, an RCT of 100 patients undergoing major surgery found that goal-directed therapy to maintain Pcv-aCO_2_ < 6 mmHg did not improve organ function compared to a standard approach [85]. Nevertheless, it led to increased dobutamine use, improved tissue oxygenation parameters, and a reduced ICU length of stay [85]. Additionally, a prospective cohort study found no association between Pcv-aCO_2_ and survival or postoperative adverse outcomes in critically ill patients following liver transplantation [86]. While the overall findings are promising, further evidence is needed to support the routine use of Pcv-aCO_2_ as a hemodynamic resuscitation target in major non-cardiac surgery patients.

##### Cardiac surgery

Clinical studies have reported conflicting findings regarding Pcv-aCO_2_ in cardiac surgery patients [87–96]. Indeed, in a prospective study of 110 cardiac surgery patients with cardiopulmonary bypass (CPB), Mukai et al. reported that postoperative Pv-aCO_2_ was independently associated with major organ morbidity and mortality (MOMM) and was the strongest predictor of MOMM, with an AUROC of 0.80 and a cut-off value of 5.2 mmHg [87]. Similarly, other observational studies have found that Pv-aCO_2_ predicted postoperative complications and was independently associated with ICU mortality [88–91].

In contrast, some studies have reported a limited ability of Pcv-aCO_2_ to predict outcomes in cardiac surgery patients [92–96]. Indeed, a retrospective study of 220 patients undergoing cardiac surgery with CPB found that Pcv-aO_2_ had poor predictive value for postoperative outcomes [92]. A recent retrospective study of 1,933 patients further confirmed the poor diagnostic performance of Pcv-aCO_2_ in predicting postoperative complications (AUROC = 0.55), despite its independent association with adverse outcomes [93]. Similarly, other observational studies have reported that Pcv-aCO_2_ did not predict major postoperative complications and was not a reliable indicator of organ dysfunction in cardiac surgery patients undergoing CPB [94–96]. These negative findings may be attributed to the effects of CPB on Pcv-aCO_2_. Indeed, CPB influences CO_2_ production, hemoglobin dilution, and pH changes, potentially altering the PCO_2_:CCO_2_ relationship. Additionally, hypothermia during surgery and subsequent rewarming can impact VCO_2_ and further affect the relationship between PCO_2_ gaps and major postoperative complications [97]. After myocardial revascularization, Pv-aCO_2_ was found to be a nonspecific parameter influenced primarily by VO_2_, body temperature, and PaCO_2_ rather than tissue perfusion [98].

A meta-analysis of 21 studies (*n* = 2,155 patients) across medical (*n* = 925), cardiovascular (*n* = 685), surgical (*n* = 483), and mixed (*n* = 62) ICUs found that elevated Pcv-aCO_2_ was associated with higher blood lactate concentrations, lower cardiac output, and reduced ScvO_2_. Additionally, in patients with shock, a high Pcv-aCO_2_ was linked to increased mortality (OR = 2.22, *p* = 0.004), but only in medical and major non-cardiac surgical patients [99]. No significant association was found between Pcv-aCO_2_ and outcomes in cardiac surgery patients. However, the meta-analysis included only two studies on cardiac surgery, making these negative findings inconclusive. As a result, the relationship between Pcv-aCO_2_ or Pv-aCO_2_ and outcomes in cardiac surgery patients remains unclear. Further studies are needed to refine its prognostic value and establish its role in clinical practice.

##### Assessment of oxygen supply to demand adequacy: effects of Dobutamine

Dobutamine, a synthetic catecholamine with strong inotropic effects due to its predominant β1-adrenergic properties [100], is commonly used to increase cardiac output and ensure adequate DO_2_ to meet tissue demands. However, beyond its systemic hemodynamic effects, dobutamine can also increase VO_2_ and VCO_2_ through its direct cellular metabolic stimulation [34, 101].

Two studies examined the effects of dobutamine titration (0 to 15 µg/kg/min) in patients with septic shock [102] and in patients with stable congestive heart failure [34]. Both studies reported a dose-dependent increase in cardiac output and DO_2_, with distinct responses at different dosage ranges [34, 102].

Between 0 and 10 µg/kg/min, dobutamine increased cardiac output and DO_2_ dose-dependently. The VCO_2_ increased, but much less than the cardiac output. This explains the commensurate decrease in Pcv-aCO_2_ attributed to increased blood flow and improved CO_2_ washout. As anticipated, SvO_2_ and ScvO_2_ improved as DO_2_ increased to organs that were still in need of oxygen. At these doses, dobutamine primarily enhances hemodynamics, improves organ perfusion, and facilitates CO_2_ clearance [34, 102]. At higher doses (10–15 µg/kg/min), while cardiac output continued to increase, VCO_2_ rose proportionally, leading to a stable Pcv-aCO_2_. This plateau effect is attributed to dobutamine’s β-adrenergic stimulation, which directly increases cellular metabolism, oxygen consumption, and CO_2_ production—a thermogenic effect observed only at these higher doses [34, 102].

Thus, Pcv-aCO₂ (or Pv-aCO₂) may reflect dobutamine-induced changes in VCO₂ and help distinguish between its hemodynamic and metabolic effects in critically ill patients [103]. Given its ease of bedside measurement and its complementary value alongside other tissue perfusion markers, Pcv-aCO₂ could serve as a useful tool for guiding hemodynamic management in the ICU.

##### Response to fluids

In a recent post-hoc analysis of a multicenter prospective study, Mallat et al. examined 205 mechanically ventilated patients with acute circulatory failure [104]. Fluid responsiveness was defined as an increase in cardiac index of > 15% following fluid administration. A post-fluid challenge decrease in Pcv-aCO_2_ ≤ 2.1 mmHg and an increase in ScvO_2_ ≥ 3.4% effectively differentiated fluid responders from fluid non-responders, with positive predictive values of 90% and 86% and negative predictive values of 58% and 64%, respectively. Additionally, both ΔPcv-aCO_2_ and ΔScvO_2_ were independently associated with fluid responsiveness in multivariable analysis. Notably, no significant relationships were found between pre-infusion ScvO_2_ or Pcv-aO_2_ levels and fluid responsiveness, which is in agreement with previous findings [105]. These findings suggest that if Pcv-aCO_2_ decreases by > 2 mmHg after a fluid challenge, clinicians can be fairly confident that the patient has responded to the fluid administration. However, if ΔPcv-aCO_2_ remains unchanged, fluid responsiveness cannot be ruled out, particularly in patients with tissue hypoxia due to the VO_2_/DO_2_ dependency phenomenon [106, 107].

Moreover, the negative predictive value of ΔScvO_2_ was poor, meaning it cannot reliably rule out the presence of fluid responsiveness [104]. Several factors may explain this limitation. Fluid administration can cause hemodilution, leading to a decrease in hemoglobin concentrations and arterial oxygen content, potentially blunting increases in ScvO_2_ [108]. Additionally, in states of VO₂/DO₂ dependency, an increase in cardiac output following fluid administration may also lead to a rise in VO₂, as the body repays its oxygen debt—thereby limiting the increase in ScvO₂ [106, 107]. However, despite its limitations in ruling out fluid responsiveness, ΔScvO_2_ demonstrated a strong positive predictive value for confirming the presence of fluid responsiveness [104]. These findings align with a recent meta-analysis of five observational studies involving 240 critically ill patients, further supporting its clinical utility [109].

In summary, while variations in Pcv-aCO₂ and ScvO₂ may serve as markers of fluid responsiveness in selected cases, their interpretation is limited in the presence of tissue hypoxia.

##### Weaning from mechanical ventilation

Weaning is typically associated with an increase in VO_2_ due to the higher work of breathing [110, 111]. An appropriate increase in cardiac output and DO_2_ is required to meet this increased metabolic demand, which may justify the use of markers of the adequacy of cardiac output to oxygen demand. Indeed, several studies have shown that a decrease in SvO_2_ during a spontaneous breathing trial (SBT) was associated with weaning and extubation failure [112, 113]. In one study, SvO_2_ progressively declined in patients who failed SBT but remained stable in those who succeeded [112]. This drop was attributed to increased oxygen extraction, likely due to the increased activity of the respiratory muscles. Similarly, Teixeira et al. [113] found that changes in ScvO_2_ from mechanical ventilation to the end of SBT were independently associated with extubation failure in patients with weaning difficulty. A decline in ScvO_2_ > 4.5% predicted extubation failure with a high positive predictive value of 93% and a negative predictive value of 90%.

Mallat et al. conducted a prospective multicenter study to assess the usefulness of this metabolic approach in predicting weaning outcomes from mechanical ventilation [114]. The study enrolled 75 patients, with extubation failure occurring in 24%. Changes in Pcv-aCO_2_ (ΔPcv-aCO_2_) and ScvO_2_ (ΔScvO_2_) during an SBT demonstrated a strong predictive value for extubation outcomes, with AUROCs of 0.865 and 0.856, respectively. However, combining ΔScvO_2_ and ΔPcv-aCO_2_ significantly improved the detection of extubation failure, yielding an AUROC of 0.940, which was superior to ΔScvO_2_ alone (*p* = 0.04) and ΔPcv-aCO_2_ alone (*p* = 0.03). Additionally, both ΔPcv-aCO_2_ and ΔScvO_2_ were independently associated with extubation failure (OR = 1.02; *p* = 0.006, and OR = 0.84; *p* = 0.02, respectively).

#### The Cv-aCO_2_/Ca-vO_2_ and Pv-aCO_2_/Ca-vO_2_ ratios and their clinical implications

Hyperlactatemia has traditionally been recognized as a marker of anaerobic metabolism secondary to inadequate cell oxygen supply [115]. In this regard, Mekontso-Dessap et al. [16] showed a significant association between the Pv-aCO_2_/Ca-vO_2_ ratio and blood lactate concentrations ≥ 2.0 mmol/L, but not between blood lactate concentrations and isolated Pv-aCO_2_ or Cv-aCO_2_ values. Nevertheless, blood lactate concentrations may increase due to many other causes than tissue hypoxia [116–118]. Remarkably, the Cv-aCO_2_/Ca-vO_2_ ratio (or its potential equivalent, the Pv-aCO_2_/Ca-vO_2_ ratio) could provide additional information to blood lactate concentrations, as non-aerobic VCO_2_ reflects abnormal cellular metabolism. Indeed, the combination of high Cv-aCO_2_/Ca-vO_2_ ratio and high blood lactate concentrations during the very early stages of resuscitation in septic shock was associated with more severe organ dysfunction and worse clinical outcomes when compared with patients attaining normal blood lactate concentrations and Cv-aCO_2_/Ca-vO_2_ ≤ 1.0 [17]. Remarkably, persistently high Cv-aCO_2_/Ca-vO_2_ ratios were related to more severe organ dysfunction and worse clinical outcomes even in patients who simultaneously attained normal blood lactate concentrations [17]. Cv-aCO_2_/Ca-vO_2_ ratio should react faster than blood lactate concentrations to short-term hemodynamic changes [13]. This makes this ratio an attractive variable to monitor, and although it is a little difficult to calculate, its interpretation is easier, with values > 1.0, suggesting ongoing anaerobic CO_2_ generation as a consequence of tissue acidosis. Thus, a high Cv-aCO_2_/Ca-vO_2_ ratio should reflect non-aerobic CO_2_ generated as a consequence of buffering of the excess of H^+^ (most of them coming from ATP hydrolysis) not recycled in the normal cell energy-generating processes (Fig. 1).

Subsequent studies corroborated the Pv-aCO_2_/Ca-vO_2_ ratio as a prognostic factor in humans with septic shock [119–121] and its relationship with regional lactate/pyruvate ratio and acute kidney injury in experimental models [122]. In addition, increases in VO_2_ following fluid loading were better predicted by both Ccv-aCO_2_/Ca-cvO_2_ and Pcv-aCO_2_/Ca-cvO_2_ ratios than by blood lactate concentrations [107] or by baseline ScvO_2_ [106], which suggests the superiority of Ccv-aCO_2_/Ca-cvO_2_ and Pcv-aCO_2_/Ca-cvO_2_ ratios over O_2_-derived variables and lactate kinetics to detect tissue hypoxia or to some extent, to detect non-aerobic VCO_2_. Furthermore, experimental data suggest that restoration of microcirculatory blood flow distribution parallels proportional normalization in the Cv-aCO_2_/Ca-vO_2_ ratio in septic shock [48, 64].

Despite the apparent equivalence between Cv-aCO_2_/Ca-vO_2_ and Pv-aCO_2_/Ca-vO_2_ ratios, conflicting results have been reported, as some authors showed that Cv-aCO_2_/Ca-vO_2_ but not Pv-aCO_2_/Ca-vO_2_ was related to adverse clinical outcomes in septic shock [17]. In contrast, others demonstrated that only Pv-aCO_2_/Ca-vO_2_ did [58]. This may be attributed to the complex nature of the PCO₂:CCO₂ relationship, which is influenced by multiple physiological factors (see limitations of Pv-aCO_2_ section).

In conclusion, high Cv-aCO_2_/Ca-vO_2_ and Pv-aCO_2_/Ca-vO_2_ ratios might reflect increased non-aerobic VCO_2_ generation during tissue hypoxia or even under normoxic conditions, but with concomitant cell acidosis.

#### Errors in PCO_2_ gap calculation

Several pre-analytical sources of error in PCO_2_ measurement must be minimized to ensure accurate interpretation of Pv-aCO_2_. These include using inappropriate sample containers, insufficient sample volume relative to the anticoagulant, and contamination from resident fluid in the line, air bubbles, or venous blood. Even with strict precautions to reduce pre-analytical and analytical errors, a prospective study by Mallat et al. [123] found that the least significant change (the minimal change required to confirm a true measurement variation) for Pcv-aCO_2_ was ± 2 mmHg. This implies that changes in Pcv-aCO_2_ must exceed ± 2 mmHg to be considered clinically significant rather than a result of natural measurement variability [123].

Overall, clinicians should remain aware of these limitations, but these factors should not discourage the clinical use of Pv-aCO₂.

## Conclusions

**Fig. 3:**
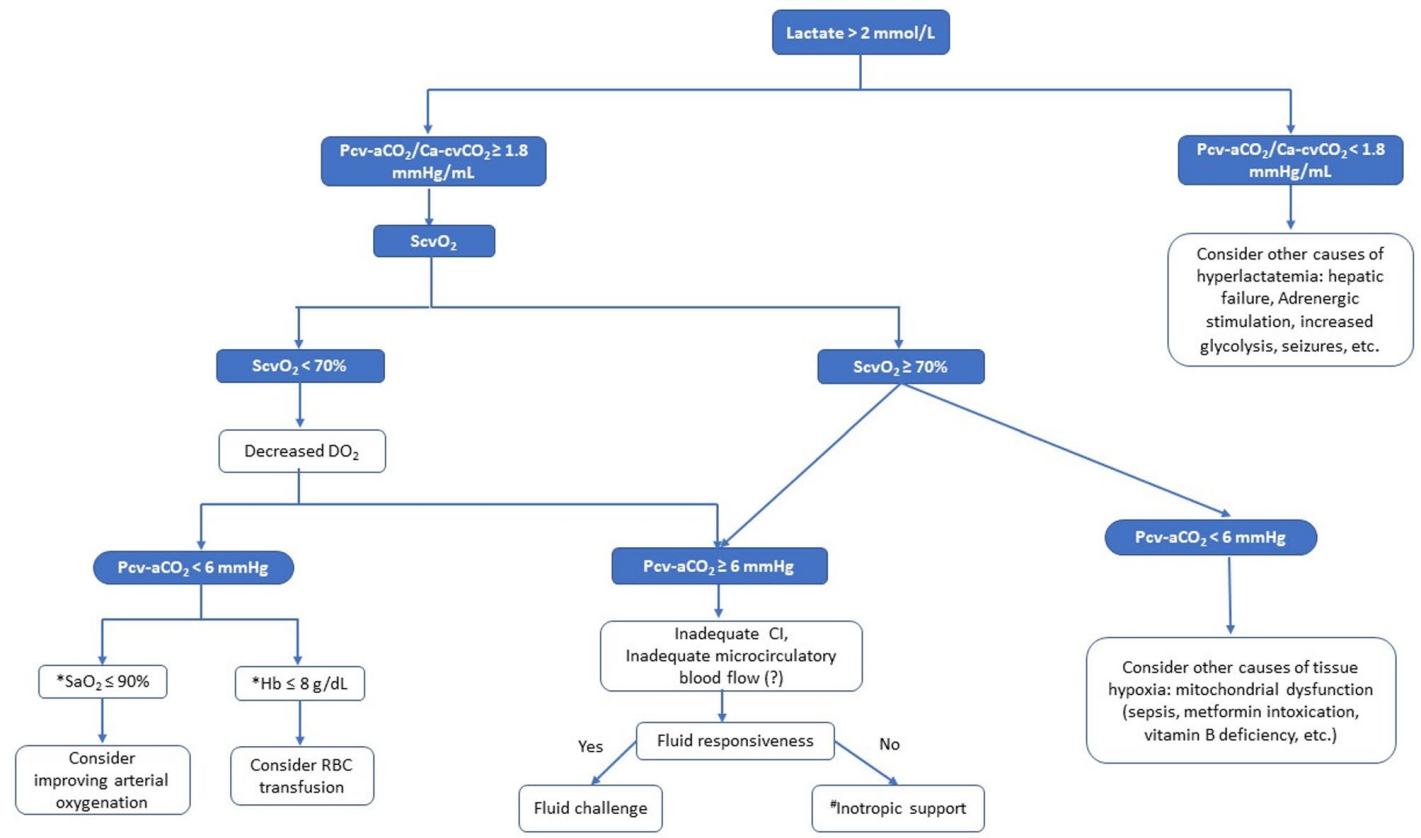
CO_2_ and O_2_-derived variables guided protocol. Proposed guided algorithm integrating lactate, central venous oxygen saturation (ScvO_2_), the central venous-to-arterial CO_2_ pressure difference (Pcv-aCO_2_), and the ratio between Pcv-aCO_2_ and the difference between arterial and central venous oxygen content (Pcv-aCO_2_/Ca-cvO_2_). SaO_2_, arterial oxygen saturation; CI, cardiac index; Hb, hemoglobin; RBC, red blood cell ^*^ SaO₂ ≤ 90% and Hb ≤ 8 g/dL are provided as general references and should be interpreted in the context of the clinical situation ^#^ We advise performing echocardiography before considering inotropic support

Tissue hypoxia occurs when cells exhibit abnormal oxygen utilization, triggering anaerobic metabolism. Early identification of tissue hypoperfusion and hypoxia is critical to preventing clinical deterioration. Various tools are available for detecting and monitoring these conditions, but many require specialized equipment that is either unavailable in most ICUs or primarily used for research purposes. Based on CO_2_- and O_2_-derived variables, the metabolic approach offers an attractive alternative, as it only requires a central venous catheter and an arterial line—devices already in place for most critically ill patients. Numerous experimental and clinical observational studies have demonstrated the reliability of Pcv-aCO_2_ and the Pcv-aCO_2_/Ca-cvO_2_ ratio in detecting tissue hypoperfusion and the presence of anaerobic metabolism (tissue hypoxia), respectively, particularly in septic shock and in postoperative non-cardiac surgery patients. Furthermore, these markers have been associated with clinical outcomes. Several diagnostic algorithms incorporating Pv-aCO_2_ have been proposed, as shown in Fig. 3. However, multicenter RCTs are needed to validate the efficacy of the metabolic approach compared to standard hemodynamic management strategies in patients with acute circulatory failure.

## Supplementary Information

Below is the link to the electronic supplementary material.

Supplementary Material 1.

## Data Availability

Not applicable.

## Abbreviations

CO_2_: Carbon dioxide
VCO_2_: Carbon dioxide production
VO_2_: Oxygen consumption
PvCO_2_: Venous carbon dioxide partial pressure
PaCO_2_: Arterial carbon dioxide partial pressure
RQ: Respiratory quotient
HCO_3_^−^: Bicarbonate
H_2_CO_3_: Carbonic acid
H_2_O: Water
DO_2_: Oxygen delivery
ATP: Adenosine triphosphate
H^+^: Hydrogen ion
CO_3_^2−^: Carbonate
Cl^−^: Chloride anion
OH^−^: Hydroxide anion
R-NH_2_: Amino groups
Hb-NH-COO^−^: Carbamino hemoglobin
CCO_2_: Carbon dioxide content
O_2_: Oxygen
PO_2_: Partial pressure of oxygen
Cv-aCO_2_: Venous to arterial carbon dioxide content difference
Ca-vO_2_: Arterial to venous oxygen content difference
Pv-aCO_2_: Venous-to-arterial carbon dioxide pressure difference
CvCO_2_: Venous carbon dioxide content
CaCO_2_: Arterial carbon dioxide content
FCD: Functional capillary density
Psl-aCO_2_: Sublingual-to-arterial CO_2_ partial pressure difference
Pmtis-aCO_2_: Gut mucosal-to-arterial PCO_2_ difference
Pgtis-aCO_2_: Gastric mucosal-to-arterial PCO_2_ differences
ScvO_2_: Central venous oxygen saturation
SvO_2_: Mixed venous oxygen saturation
OR: Odds ratio
RCT: Randomized controlled trial
ICU: Intensive care unit
SOFA: Sequential organ failure
AUROC: Area under the receiver operating curve
MOMM: Major organ morbidity and mortality
CPB: Cardiopulmonary bypass
SBT: Spontaneous breathing trial
