# Supplementary material for: Use of CO2-derived variables in critically ill patients
Source: Ann Intensive Care. 2025 Sep 25;15:142. doi: 10.1186/s13613-025-01569-2 (PMC12463780; doi:10.1186/s13613-025-01569-2)
Supplement: Supplementary file 1 — Supplementary Material 1. [file 13613_2025_1569_MOESM1_ESM.docx]

**Reviewers**

**Reviewer #1**

**Reviewer:** This is a nice extensive review of using blood CO2 levels to assess cellular metabolism The issues are well described.

**Response:** We sincerely thank the reviewer for this positive and encouraging feedback.

**Reviewer:** However, the authors ignore the original work by Dr. Max Harry Weil who used increases in end-tidal CO2 to validate blood flow in CPR and appropriate response to shock resuscitation.

**Response:** We appreciate the reviewer’s comment and acknowledge the seminal work by Dr. Max Harry Weil, who demonstrated that increases in end-tidal CO₂ correlate with improved blood flow during CPR and effective response to shock resuscitation (Crit Care Med. 1985;13(11):907–909; etc.). However, as our review focuses specifically on the venous-to-arterial PCO₂ gap and the ratio of this gap to the arterial-venous oxygen content difference, we did not include studies centered on end-tidal CO₂, which represents a distinct physiological parameter. Given the already extensive scope of the review, we aimed to maintain clarity and avoid introducing potentially tangential concepts. We hope this explanation adequately addresses the reviewer’s concern.

**Reviewer:** Indeed, Laurent Brochard showed that increases in End-tidal CO2 in response to PLR predicted fluid responsiveness.

**Response:** We thank the reviewer for the comment. However, the work referred to was not conducted by Prof. Laurent Brochard, but by Prof. Xavier Monnet and colleagues, as published in Intensive Care Medicine in 2013 (Monnet X, Bataille A, Magalhaes E, Barrois J, Le Corre M, Gosset C, Guérin L, Richard C, Teboul JL. End‑tidal carbon dioxide is better than arterial pressure for predicting volume responsiveness by the passive leg raising test. Intensive Care Med. 2013;39(1):93–100). Unless the reviewer is referring to a different study, which we may have overlooked, we would be grateful if the corresponding reference could be provided for clarification. As explained in our previous response, our review focuses specifically on the venous-to-arterial PCO₂ gap and the ratio of this gap to the arterial-venous oxygen content difference. For this reason, we did not include studies centered on end-tidal CO₂, which represents a different physiological parameter. Given the already extensive scope of the review, we aimed to maintain clarity and avoid introducing tangential concepts. We hope this explanation satisfactorily addresses the reviewer’s concern.

**Reviewer:** Also there is a very large literature on direct measures of tissue CO2 as a measure to local blood flow. These things need to be briefly stated, but do not need to be reviewed, as that is not the thrust of the paper.

**Response:** We thank the reviewer for this comment. As noted, we have already addressed the tissue-to-arterial PCO₂ gap briefly in the following sections of our review: “Can the Pv–aCO₂ be used as a marker of tissue hypoxia?”, “Tissue-to-arterial CO₂ gaps, Pv–aCO₂, and microcirculatory blood flow alterations”, and “What is the difference between tissue PCO₂ and venous PCO₂?”. We fully agree with the reviewer on the existence of a substantial body of literature regarding direct measurements of tissue CO₂ as a marker of local blood flow. However, as the reviewer also acknowledged that this was not the primary focus of our paper, we believe that expanding this topic further could detract from the central message and potentially confuse readers. Given the already broad scope of the review, we sought to maintain clarity and avoid introducing tangential discussions. We hope this explanation adequately addresses the reviewer’s concern.

**Reviewer #2**

**Reviewer:** This manuscript reminds the reader of physiological concepts governing CO2 production, its content in blood, its transport, its elimination and its relationship with blood flow. It provides relevant information regarding clinical applications.

**Response:** We sincerely thank the reviewer for this positive and encouraging feedback.

**Reviewer:** Page 7 (CO2 and related compounds in blood): This text regarding bicarbonate doesn't make sense to me: 'the proportion (of HCO3-) is lower in venous blood because of the Haldane effect'. Shouldn't it be the opposite? In essence, the Haldane effect describes deoxygenated hemoglobin binding CO₂ more readily than oxygenated hemoglobin. HCO3- in venous blood is higher than in arterial blood.

**Response:** We thank the reviewer for this important point and apologize if our original wording was unclear. The reviewer is correct that the absolute concentration of HCO₃⁻ is higher in venous blood than in arterial blood. This is because tissues produce CO₂ as a metabolic byproduct, increasing total CO₂ content in venous blood. The Haldane effect explains why a greater proportion of this CO₂ is carried in venous blood as carbamino compounds—deoxygenated hemoglobin binds CO₂ more readily and buffers protons more effectively. Consequently, although the absolute HCO₃⁻ concentration is higher in venous blood, its proportion relative to total CO₂ is slightly lower than in arterial blood due to this increased carbamino carriage. We have revised the statement in the manuscript to read: “In arterial blood, bicarbonate (HCO₃⁻) accounts for approximately 90% of the total CO₂ content (i.e., ≈ 435 ml/L in whole blood). Although the absolute HCO₃⁻ concentration is slightly higher in venous blood due to increased CO₂ loading, the proportion of total CO₂ carried as HCO₃⁻ is slightly lower. This is because a greater fraction of CO₂ is bound to hemoglobin as carbamino compounds in venous blood—a shift facilitated by the Haldane effect.” Changes in the manuscript are highlighted in red. We hope this clarification adequately addresses the reviewer’s concern.

**Reviewer:** I think this important section requires some re-writing for clarity: 'Because of the CO2 dissociation curve slope, PCO2 must increase from 40 mmHg in arterial blood to only 46 mmHg in mixed-venous blood to increase the total CO2 content by ≈ 4 mL/dL (from 48 to 52 mL of CO2 gas/dL), which is required to remove the CO2 generated by aerobic mitochondrial working. Accordingly, without the Haldane effect, the venous PCO2 would increase significantly more for similar increases in CCO2.' This section doesn't make sense to me, especially when written within the context of the Haldane effect. The CO2 dissociation curve is not the cause of the increase of PCO2 in mixed venous blood compared to arterial blood. The cellular production of CO2 (and the Haldane effect) is the cause of this increase, and that rate of increase can be plotted on CO2 dissociation curve. Please check that the increase from 48 to 52ml of CO2 is CO2 gas - I would have thought that it was all CO2, not just CO2 gas. I also did not follow why CO2 would have to increase to remove CO2 generated by 'aerobic mitochondrial working' (sic). Finally, I did not understand why the PCO2 would have to increase significantly for more similar increases in CCO2.

**Response:** We thank the reviewer for these important points and apologize if our original wording was unclear. Below are the responses to the different points raised by the reviewers:

- This section doesn't make sense to me, especially when written within the context of the Haldane effect. The CO2 dissociation curve is not the cause of the increase of PCO2 in mixed venous blood compared to arterial blood. The cellular production of CO2 (and the Haldane effect) is the cause of this increase, and that rate of increase can be plotted on CO2 dissociation curve.

Response: We agree with this reviewer’s comment. The increase in PCO2 is due to tissue CO2 production. The dissociation curve merely describes the relationship between PCO2 and CCO2 content, not the cause of PCO2 elevation. We have rephrased the statement in the manuscript.

- Please check that the increase from 48 to 52ml of CO2 is CO2 gas - I would have thought that it was all CO2, not just CO2 gas.

Response: We agree with the reviewer that the value refers to total CO₂ content rather than CO₂ gas alone. We have corrected the terminology accordingly in the manuscript and thank the reviewer for drawing our attention to this oversight.

- I also did not follow why CO2 would have to increase to remove CO2 generated by 'aerobic mitochondrial working' (sic).

Response: We agree with the reviewer’s comment and apologize for the unclear phrasing. Our intention was to convey that the CO₂ content in blood increases as a consequence of aerobic metabolism, which generates CO₂ as a byproduct. We thank the reviewer for highlighting this ambiguity. We have rephrased the statement in the manuscript.

- Finally, I did not understand why the PCO2 would have to increase significantly for more similar increases in CCO2.

Response: We agree that this point needed clarification. The Haldane effect facilitates CO₂ carriage in deoxygenated blood, so in its absence, a greater rise in PCO₂ is required to achieve the same CCO₂, especially at higher levels of oxygenation.

Based on all these comments we have rephrased the statement in the manuscript as follows: “Aerobic mitochondrial metabolism generates CO₂, increasing CCO₂ by ≈ 4 mL/dL from arterial to mixed venous blood (from 48 to 52 mL/dL), with a small PCO₂ rise (from 40 mmHg in the arterial blood to 46 mmHg in the mixed venous blood) due to the steep CO₂ dissociation curve. Without the Haldane effect, which enhances CO₂ carriage in deoxygenated hemoglobin, venous PCO_2_ would rise significantly more for similar CCO_2_ increases because less CO₂ could be stored as carbamino compounds and as bicarbonate.” Changes in the manuscript are highlighted in red. We hope this clarification adequately addresses the reviewer’s concern.

**Reviewer:** Please also consider rephrasing this paragraph and the legend to Figure 2 for clarity:

'Remarkably, the relationship between PCO2 and CCO2 is also influenced by variations in H+ concentration, temperature, and hematocrit. Thus, blood CCO2 decreases as pH and HCO3 also do, while an increase in temperature reduces the CO2 solubility, deviating the CCO2:PCO2 relationship downwards (Figure 2).'

Figure 2A shows three CO2 dissociation curves - 2 curves are at relatively close HbO2 intervals (98% and 75%) then an extreme, at 0% HbO2. The authors write that the figure illustrates that the 'differences in the curves result in higher CCO in blood'. I cannot see that this is illustrated by figure 2A. In fact, the CO2 dissociation curves at 75% HbO2 and 98% HbO2 are almost parallel and the CCO2 increases almost parallel also - so there is no effect of the 'curve'. The effect 'due to the curves' are only apparent at 0% HbO2 which is unphysiological.

**Response:** Thank you very much for your comment. Figure 2, Panel A, depicts the classic relationship between PCO_2_ and CCO_2_ at different levels of hemoglobin oxygen saturation, including a theoretical completely non-oxygenated hemoglobin (i.e., SO_2_ = 0%), such as it has been previously illustrated in textbooks and manuscripts referring to the Haldane effect. Such a diagram is perfectly reproducible under controlled (experimental) conditions, whereby we assumed the reviewer refers to “ex vivo” or “experimental” conditions. Consequently, and trying to avoid confusion, we adapted the figure legend accordingly. In addition, we also decided to delete the phrase about the CCO_2_ that you are mentioning. However, it is worth mentioning that when looking at the curve, the lower Hb oxygen saturation, the higher CCO_2_ for similar PCO_2_ values. And of course, “there is NOT EFFECT of the curve”, because the curve itself only depicts the total phenomenon linked to hemoglobin oxygen saturation. We hope this clarification adequately addresses the reviewer’s concern.

**Reviewer:** Further, the authors write 'Thus, blood CCO2 decreases as pH and HCO3 also do, while an increase in temperature reduces the CO2 solubility, deviating the CCO2:PCO2 relationship downwards.'. Presumably the authors are referring to figure 2B here. The figure does not illustrate changes in pH and HCO3, only base excess. While I understand that BE is related to HCO3-, it is also dependent on pH. In real life, there may be situations where BE is not wholly dependent on HCO3-, and Figure 2B may not be completely accurate. I suggest plotting the CO2 dissociation curves for changes in HCO3- for accuracy.

**Response:** Thank you very much for your comment. Figure 2, Panel A, depicts a classic relationship between hydrogen ion accumulation (using base excess as an equivalent) and the PCO_2_:CCO_2_ relationship. We respectfully disagree with the reviewer suggesting that depicting HCO_3_ isopleths in the relationship CCO_2_:PCO_2_ would be more accurate. All variables determining pH according to the Henderson-Hasselbalch approach show a high degree of mathematical coupling (i.e., CO_2_, H_2_CO_3_, and HCO_3_), so BE isopleths could be more appropriate to describe the phenomenon linked to hydrogen ion accumulation (although admittedly, some coupling also exists when using this approach). We hope this clarification adequately addresses the reviewer’s concern.

**Reviewer:** Page 11. Re paragraph on 'Can the Pv-aCO2 be used as a marker of tissue hypoxia?' This is an important section in the manuscript. I suggest some language edits for clarity and conciseness.

**Response:** We appreciate the reviewer’s comment. We have revised the paragraph for clarity and conciseness, with changes highlighted in red in the manuscript. We trust that the revised version improves readability and addresses the reviewer’s concern.

**Reviewer:** I appreciate the data presented to indicate that Pv-aCO2 is dependent on flow rather than oxygenation.

**Response:** We sincerely thank the reviewer for this positive and encouraging feedback.

**Reviewer:** I would add in this section something about the interchangeability (or lack of) between mixed and central PCO2 gap (e.g. van Beest ICM2013) although I note that this is already mentioned in the section on septic shock.

**Response:** We appreciate the reviewer’s comment; however, we respectfully disagree with adding a discussion on the interchangeability between mixed and central PCO_2_ gaps in this section. The aim here is to address the general concept of the PCO­_2_ gap and its potential relationship with tissue hypoxia. Adding details about the interchangeability between mixed and central PCO_2_ gaps would go beyond the scope of this section, could distract from the main message, and might make the section harder to follow. As the reviewer noted, we have already addressed this point briefly in another section of the manuscript. We hope this explanation satisfactorily addresses the reviewer’s concern.

**Reviewer:** I would also mention the effects of ventilation on the PCO2 gap (e.g. Guo et al. Eur J Med Res 2020). I note that the latter was presented in the limitations section, but I suggest that it be moved here for better flow.

**Response:** We appreciate the reviewer’s comment; however, we respectfully disagree that relocating the discussion on the effects of ventilation on the PCO_2_ gap to this section would improve the flow of the text. As noted in our previous response, the purpose of this section is to present the general concept of the PCO_2_ gap and its potential relationship with tissue hypoxia. Introducing details on the effects of ventilation here would extend beyond the intended scope, potentially diverting attention from the main message and making the section less cohesive. As the reviewer mentioned, this topic is already covered in the limitations section, within a dedicated sub-paragraph, which has now been moved to be placed earlier in the manuscript under “Other factors influencing PCO_2_ gap” as requested by reviewer # 3. We hope this clarification addresses the reviewer’s concern.

**Reviewer:** Please add the study by Guo for completeness.

**Response:** We thank the reviewer for this comment. We have added the requested reference to the manuscript as follows: “These findings regarding the effects of hyperventilation on the Pcv-aCO_2_ were later confirmed by Guo et al. (63)”. Changes are highlighted in red. We hope this clarification satisfactorily addresses the reviewer’s concern.

**Reviewer:** Page 12. Excellent subsection on the effect of microcirculatory flow and Pv-aCO2. Can you state whether or not these were independent of macrocirculatory flow?

**Response:** We thank the reviewer for this important comment. The studies by Créteur et al. (ref 44) and Nevière et al. (ref 45) did not assess whether the relationship between microcirculatory flow and Pv-aCO_2_ is dependent on cardiac output. In contrast, Ospina-Tascón et al. (ref 17) reported that the effect of microcirculatory flow on Pv-aCO_2_ was independent of cardiac output, as stated in the manuscript (page 12): “Increases in Pv-aCO_2_ were paralleled by a gradual decrease in sublingual functional capillary density and increased microvascular flow heterogeneity, independently of cardiac output variations (17).” In the study by Mesquida et al. (ref 46), however, this relationship was dependent on cardiac output. Notably, in that study microcirculation was assessed using near-infrared spectroscopy, a technique that does not directly measure microcirculatory blood flow. We have now added the following clarification to page 13 of the manuscript: “However, in that study, microcirculation was evaluated using near-infrared spectroscopy, a technique that does not directly measure microcirculatory blood flow (46).” Changes are highlighted in red. We hope this revision satisfactorily addresses the reviewer’s concern.

**Reviewer:** Page 14. 'The venous-arterial PCO2 to arterial-venous O2 content ratio (Cv-aCO2/Ca-vO2 and Pv-aCO2/CavO2 ratios)'. The heading describes the Pv-aCO2/Ca-vO2 ratio but in brackets both the Pv-aCO2/Ca-vO2 ratio and the Cv-aCO2/Ca-vO2 ratios are given. Please correct.

**Response:** We thank the reviewer for this comment and apologize for the oversight. We have corrected the heading to read: “Venous-arterial PCO and CO_2_ content to arterial-venous O_2_ content ratio (Cv-aCO_2_/Ca-vO_2_ and Pv-aCO_2_/Ca-vO_2_ ratios).” We hope this revision satisfactorily addresses the reviewer’s concern.

**Reviewer:** The section 'Clinical relevance of PCO2 gaps' is well-written and I do not have much too add except to recommend some language edits.

**Response:** We sincerely thank the reviewer for the positive and encouraging feedback. We have revised the section to improve the language and clarity.

**Reviewer:** The limitations of PCO2-derived indices are important. However, much of what is written is already covered in previous sections. I would delete this and incorporate limitations under the relevant sections. There is also a lot of repetition, for example, the Haldane effect and acidosis are already covered in previous sections.

**Response:** We appreciate the reviewer’s comment and agree that some limitations of the PCO_2_ gap were mentioned briefly in earlier sections. In the limitations section, however, these points were addressed more comprehensively. While some repetition is unavoidable in a scoping review, we do not believe these limitations can be fully integrated into the earlier sections without losing context. Nevertheless, as suggested by Reviewer #3, we have moved most of the limitations section to an earlier part of the manuscript, under the heading “Other factors that influence the PCO_2_ gap.” We hope we have addressed the reviewer’s concern appropriately.

**Reviewer:** Minor: Page 7. There is a typo with the numbering (there are two '2's).

**Response:** We thank the reviewer for this comment and apologize for this typo, which we have now corrected in the manuscript.

**Reviewer:** P10. Suggest 'no flow' instead of 'stopped blood flow'.

**Response:** We thank the reviewer for this comment. We have made the requested correction in the manuscript to read: “no blood flow.”

**Reviewer:** P11. Regarding the Vallet et al JAP200) experiments: 'Comparable decreases in DO2 and VO2 were induced by progressive reductions of the inspired O2 fraction (hypoxic hypoxia) but preserving flow velocity and by gradual reductions of the roller pump velocity (ischemic hypoxia) but maintaining FiO2.' Suggest a minor edit to 'To examine the isolated effects of hypoxia or flow, decreases in DO2 and VO2 were induced by progressive reductions of the inspired O2 fraction but preserving flow (hypoxic hypoxia) and by gradual reductions of the roller pump velocity but maintaining FiO2 (ischemic hypoxia)'.

**Response:** We thank the reviewer for this comment. We have made the suggested modification in the manuscript.

**Reviewer:** P11. '...Pmtis-aCO2 depicted a slight increase...' . Suggest minor edit: '...Pmtis-aCO2 increased slightly....'.

**Response:** We thank the reviewer for this comment. We have made the suggested modification in the manuscript.

**Reviewer:** P11. Similarly 'Consequently, a normal Pv-aCO2 might not discard tissue hypoxia or dysoxia' requires a language edit.

**Response:** We thank the reviewer for this comment. We have revised the sentence for clarity, which now reads: “Consequently, a normal Pv-aCO_2_ does not rule out tissue hypoxia or dysoxia.” We hope this revision satisfactorily addresses the reviewer’s concern.

**Reviewer:** P12. 'thus supporting the leading role of microvascular blood flow on gastric-tissue CO2 accumulation.' I would tone this down, we do not know if microcirculatory blood flow plays a 'leading role'.

**Response:** We thank the reviewer for this comment. We have revised the statement as requested, which now reads: “suggesting that microvascular blood flow may play an important role in gastric-tissue CO_2_ accumulation.” We hope this revision satisfactorily addresses the reviewer’s concern.

**Reviewer:** P13. 'Schlichtig et al. have shown that tissue PCO2 in the gastric mucosa reached levels comprised between 90 and 210 mmHg...'. Remove 'comprised'.

**Response:** We have now deleted “comprised” from the sentence as requested by the reviewer.

**Reviewer:** P14. '...VCO2 decreased slightly less than the VO2 falls, with the subsequent increase in the VCO2/VO2 ratio' to 'VCO2 decreased less than the VO2 decrease, with the subsequent increase in the VCO2/VO2 ratio..'

**Response:** We thank the reviewer for this comment. We have now corrected the statement as requested by the reviewer.

**Reviewer:** P15. 'Nevertheless, at such extremely low hemoglobin values (around 2 g/dL almost never encountered in clinical settings), inherent measurement errors might be amplified in the total Pv-aCO2/Ca-vO2 ratios, while indirect calorimetry assessment could be unappropriated for the highly variable timing to equilibrate different CO2 stores (CO2 dissolved in plasma, bicarbonate, and CO2 in tissues) during induction and resuscitation of shock. In addition, PCO₂ can rise sharply at extremely low hematocrit levels, with minimal changes in total CCO₂, due to impaired CO₂ buffering and binding capacity resulting from reduced hemoglobin (53).' Requires a language edit.

**Response:** We thank the reviewer for this comment. We have now edited the statement, which now reads: “Nevertheless, at extremely low hemoglobin levels (~2 g/dL, rarely seen in clinical practice), inherent measurement errors may be amplified in the total Pv-aCO_2_/Ca-vO_2_ ratio. In such conditions, indirect calorimetry may also be inappropriate due to the highly variable time required for equilibration among different CO_2_ stores (CO_2_ dissolved in plasma, bicarbonate, and tissue CO_2_) during the induction and resuscitation of shock. Furthermore, PCO_2_ can rise sharply at very low hematocrit levels, with minimal changes in total CCO_2_, because reduced hemoglobin impairs CO_2_ buffering and binding capacity (53).” We hope this revision satisfactorily addresses the reviewer’s concern.

**Reviewer:** P18. Change 'Major general surgery' to major 'non-cardiac surgery'.

**Response:** We thank the reviewer for this comment. We have now corrected the heading as requested by the reviewer.

**Reviewer:** P19. 'Similarly, other observational studies reported that Pcv-aCO2 did not predict major postoperative complications, which was not a reliable indicator for predicting organ dysfunction in cardiac surgery patients with CPB'. Requires language edit.

**Response:** We thank the reviewer for this comment. We have now edited the statement, which now reads: “Similarly, other observational studies have reported that Pcv-aCO_2_ did not predict major postoperative complications and was not a reliable indicator of organ dysfunction in cardiac surgery patients undergoing CPB.” We hope this revision satisfactorily addresses the reviewer’s concern.

**Reviewer:** The manuscript comprehensively brings together current knowledge about PCO2-derived indices. The authors should be congratulated for conducting such a thorough review based on their own work and others.

**Response:** We sincerely thank the reviewer for the positive and encouraging feedback.

**Reviewer:** The manuscript is lengthy and repetitive at times, and would benefit from language editing for clarity and stringency in writing. I have made a few suggestions, but these are non-exhaustive.

**Response:** We thank the reviewer for this comment. We have revised the manuscript for clarity and conciseness, incorporating the reviewer’s suggestions. While we acknowledge that the manuscript is lengthy and contains some repetition, this is often unavoidable in scoping reviews given their comprehensive nature. We have, nevertheless, made efforts to minimize redundancy where possible. We hope these revisions satisfactorily address the reviewer’s concern.

**Reviewer #3**

**Reviewer:** A perfect and complete overview of the (path)physiology and clinical implications and use of increased dPCO2.

**Response:** We sincerely thank the reviewer for the positive and encouraging feedback.

**Reviewer:** I have a few suggestions to improve the manuscript a bit. Introduction: The discussion about the use of lactate to resuscitate patients is very correct. But I think the authors could include a more general comment about the use of lactate in that it always normalizes in patients that survive. And so, lactate, while not very helpful in the early resuscitation, could be seen as an overall marker of the efficacy of treatment and the correction of the pathophysiology involved in the dynamics of lactate levels. Over the many years lactate levels (and it's dynamics) have almost always been associated with morbidity and mortality even in different clinical contexts (Vincent et al. Crit Care 2016(1):257). Nevertheless, the authors, later on, provide relevant studies showing that improvements in the dPCO2 also related to faster decreases/normalization of lactate levels. This all is of course related to the fact that in definitions and studies related to shock an increased lactate is used as a mandatory element.

Also, see my comments to Figure 3.

So maybe rephrase this part of the introduction to more clearly describe the differences but also the fact that lactate reflects much more than tissue hypoperfusion/hypoxia.

**Response:** We appreciate the reviewer’s comments. We believe that much of the feedback is already addressed in the current manuscript, where we state: “Other markers, such as blood lactate concentration, have been demonstrated to be useful in detecting more severe cases and in guiding resuscitation during shock states (6–8). Indeed, lactate-guided therapy significantly reduced hospital mortality in critically ill patients when adjusting for predefined risk factors (9). However, blood lactate concentration may increase for many reasons unrelated to anaerobic metabolism (10, 11). In addition, its dynamics is too slow for both an increase during shock progression and a decrease during the resuscitation phase (12). Furthermore, blood lactate concentration relies on the balance between lactate generation and its reuptake by some tissues such as the liver and kidneys. Altogether, these limitations make lactate an imperfect marker of tissue anaerobic metabolism and a guide for resuscitation.” In this section, we already note that lactate changes slowly, that it depends on the balance between production and clearance (reflecting the underlying pathophysiology), and that it is an imperfect marker of anaerobic metabolism.

As requested by the reviewer, we have now added: “In survivors, lactate levels almost invariably return to normal, and lactate kinetics have consistently been associated with morbidity and mortality across diverse clinical contexts (Vincent et al. Crit Care 2016;20:257).” We have also modified the last sentence to read: “Altogether, these limitations make lactate an imperfect marker of tissue anaerobic metabolism, tissue hypoperfusion, and a standalone guide for resuscitation.” Changes are highlighted in red. We hope this revision satisfactorily addresses the reviewer’s concern.

**Reviewer:** Page 6-line 29: '……then increasing RQ, even under preserved systemic oxygen delivery (DO2) (21)" Not clear what is meant by this part of the sentence.

**Response:** We thank the reviewer for this comment and apologize for the confusing wording. Our intended meaning was that, in cases of mitochondrial blockage, aerobic VO_2_ and VCO_2_ both decrease; however, the fall in VCO_2_ is less pronounced due to anaerobic CO_2_ production, leading to an increased RQ even when oxygen delivery to the tissues is preserved. We have rephrased the sentence in the manuscript as follows: “Remarkably, non-aerobic CO_2_ generation has been demonstrated in experimental metformin intoxication, in which lactic acidosis was accompanied by a non-proportional decrease in VCO_2_ relative to the concomitant VO_2_ fall (due to mitochondrial blockage by metformin), resulting in an increase in RQ even under preserved systemic oxygen delivery (DO_2_).” We hope this revision satisfactorily addresses the reviewer’s concern.

**Reviewer:** Page 11-line 10: "…effects of hypoxic vs. ischemic hypoxia…". This might be confusing for the reader who is not very much into this topic. Ischemia refers to a problem in oxygen delivery, where perfusion is the main cause but not the only one as hypoxia is also a problem in oxygen delivery as oxygen content decreases. What Vallet et all compared is hypoxemia/low oxygen saturation (thus low oxygen content) to a state of decreased flow with normal oxygen content. So, although Vallet uses the same terminology in the title of his manuscript, the authors can maybe replace this with the terminology they use later: hypoxic hypoxia versus ischemic (perfusion) hypoxia.

**Response:** We thank the reviewer for this comment. This paragraph has already been revised as requested by Reviewer #2, which also addresses your concern. It now reads: “To examine the isolated effects of hypoxia or flow, decreases in DO_2_ and VO_2_ were induced by progressive reductions of the inspired O_2_ fraction (FiO_2_) while preserving flow (hypoxic hypoxia) and by gradual reductions of the roller pump velocity while maintaining FiO_2_ (ischemic hypoxia).” We hope this revision satisfactorily addresses your comment.

**Reviewer:** Page 13- What is the difference between tissue and venous PCO2

The problem is that a local PCO2 is almost by definition not strongly related to the total effluent of the regional circulation. So, comparing gastric mucosal PCO2 (at a very small area in the stomach) to portal vein PCO2 assumes that the hypoperfusion detected in that area of the gastric mucosa is equivalent/reflecting the hypoperfusion in the whole GI region. With the observed differences in the PCO2 levels it is most certainly not the case as otherwise the portal vein would have a much higher PCO2 as well. So maybe the authors can rephrase this part to underscore that a local increased PCO2 does not reflect hypoperfusion in the whole regional circulation.

**Response:** We thank the reviewer for this insightful comment. We agree that a local PCO_2_ measurement—such as gastric mucosal PCO_2_—reflects conditions in a very limited area and may not represent the overall status of the entire regional circulation. We have rephrased the relevant part of the manuscript to emphasize that an increase in local tissue PCO_2_ might not reflect hypoperfusion in the entire regional circulation, by adding: “However, a locally increased PCO_2_ in the gastric mucosa might not reflect hypoperfusion in the entire regional circulation.” We hope this modification satisfactorily addresses the reviewer’s concern.

**Reviewer:** This patchy hypoperfusion in the microcirculation was also addressed by Ince et al when studying the effect of different shock models on the PO2 gap between tissue and venous effluent related to patchy microcirculatory perfusion (Crit Care Med 1999;7:1369).

**Response:** We thank the reviewer for this insightful comment and fully agree. However, as noted in the manuscript, oxygen diffusibility is more limited than that of CO_2_. Consequently, CO_2_ can diffuse over longer distances than O_2_ and still reach the venous side even under very low-flow conditions, including situations where some microvascular areas are almost non-perfused.

**Reviewer:** Page 15-line 29 "….could identify the presence of non-aerobic VCO2 mediated or not by tissue hypoxia (mitochondrial dysfunction)…" The use of tissue hypoxia while adding (mitochondrial dysfunction) is somewhat confusing as the authors describe tissue hypoxia due to many other factors as well. So maybe chance in a more descriptive sentence.

**Response:** We thank the reviewer for this comment and agree that the original wording could be confusing. In the manuscript, we have defined tissue hypoxia as limited tissue O_2_ availability. To avoid ambiguity, we have rephrased the sentence to distinguish between anaerobic CO_2_ production due to limited O_2_ availability and that due to mitochondrial dysfunction, which can occur even when O_2_ delivery is preserved. The sentence now reads: “…could identify the presence of non-aerobic VCO_2_, whether mediated by tissue hypoxia (limited O_2_ availability) or mitochondrial dysfunction (with preserved O_2_ availability).” We hope this modification satisfactorily addresses the reviewer’s concern.

**Reviewer:** Page 15 - Clinical relevance: In the following paragraphs the authors describe the usefulness of dPCO2 in different patient groups. But from page 20 on it seems to refocus on therapeutic interventions describing the effects of dobutamine and fluids. Followed by weaning from mechanical ventilation. Where weaning seems to point to clinical relevance, dobutamine and fluids seem to focus on treatment. This is a bit confusing. So maybe create a paragraph on treatment just like the authors provide in Figure 3.

**Response:** We thank the reviewer for this comment. We consider the subsections “Assessment of oxygen supply-to-demand adequacy: effects of dobutamine” and “Response to fluids” to be integral to the overall Clinical relevance section. The intent of the dobutamine subsection is to illustrate how the PCO_2_ gap can assist in titrating dobutamine doses, since it reflects the adequacy of oxygen supply relative to demand; used alongside other parameters, such as venous oxygen saturation, it may help optimize therapy. Similarly, the Response to fluids subsection demonstrates how the PCO_2_ gap can serve as a marker of fluid responsiveness in specific contexts. In the Septic shock subsection, we also discuss the potential role of the PCO_2_ gap in guiding hemodynamic resuscitation, and in other sections we highlight its association with postoperative complications and poor outcomes, suggesting that targeting the PCO₂ gap might improve outcomes in major non-cardiac surgery. Thus, potential therapeutic implications are addressed throughout the Clinical relevance section. We do not believe that creating a separate treatment paragraph to explain Figure 3 would add value, as the figure is self-explanatory and such a paragraph would be redundant. We hope that we have addressed the reviewer’s concern appropriately.

**Reviewer:** Page 25-Limitations of PCO2 gaps

This is a more (patho)physiologic discussion of the dPCO2 and could maybe be better placed earlier in the manuscript especially as the authors already refer to these elements earlier on. When placed earlier maybe change the title of this paragraph as it is not a limitation but rather other factors that influence the dPCO2.

**Response:** We thank the reviewer for this insightful comment. Following the suggestion, we have moved most of the former Limitations section to an earlier part of the manuscript under the title “Other factors influencing the PCO_2_ gap.” The Limitations section has been replaced with a new section titled “Errors in PCO₂ gap calculation.” We hope this modification satisfactorily addresses the reviewer’s concern.

**Reviewer:** Figure 3. This is a very nice extension of the flowchart proposed by Hernandez et al (Intensive Care Med. 2019;45(1):82-5) as it adds elements of oxygen content. In contrast to that flowchart what is missing in the current one is that the uses should also think about other possible sources of increases lactate (metformin, vitamin B, lymphoma, AIDS medication etc).

**Response:** We sincerely thank the reviewer for the positive and encouraging feedback. We agree that there are many potential causes of elevated lactate levels. Several are already noted in Figure 3; for example, under the case “Pcv-aCO_2_ < 6 mmHg,” we include “Consider other causes of tissue hypoxia: mitochondrial dysfunction (sepsis, metformin intoxication, etc.).” The examples mentioned by the reviewer, such as vitamin B deficiency (beriberi) and AIDS medications, increase lactate via mitochondrial dysfunction (already noted in Figure 3), while lymphoma does so primarily through increased aerobic glycolysis (Warburg effect). Increased aerobic glycolysis is already noted in Figure 3 under “Consider other causes of hyperlactatemia” in the “Pcv-aCO_2_/Ca-cvO_2_ < 1.8 mmHg/mL” case. While it is not feasible to list all possible causes of hyperlactatemia in the figure, we have added “vitamin B deficiency” to address the reviewer’s suggestion. We hope this revision satisfactorily addresses the reviewer’s concern.

**Reviewer:** Figure 3. In addition, the authors also use the starting point as the patient with an increased lactate level so (just like Hernandez et al) using an increased lactate level as a potential representation of tissue hypoperfusion/hypoxia.

This of course raises the question: what if you have a patient with a normal lactate and (for instance) hypotension (so at least some sign of circulatory dysfunction). Should one measure ScvO2, dPCO2 etc to rule out tissue hypoperfusion?

**Response:** We thank the reviewer for this insightful comment and fully agree with the point raised. Tissue hypoperfusion can indeed occur despite normal lactate levels. Therefore, in the presence of signs of acute circulatory failure (e.g., hypotension, prolonged capillary refill time, mottled skin) and normal lactate, we recommend measuring ScvO_2_ and Pcv-aCO_2_ to help guide hemodynamic resuscitation. Outcomes are generally better when lactate is normal. Our algorithm in Figure 3 begins with elevated lactate, as this often indicates possible tissue hypoxia and is typically associated with poor prognosis. In such cases, the next step is to confirm or exclude tissue hypoxia using the Pcv-aCO_2_/Ca–cvO_2_ ratio. If tissue hypoxia is present, determining its mechanism becomes the priority, for which ScvO_2_ and Pcv-aO_2_ measurements are useful. We hope that we have addressed the reviewer’s concern appropriately.

Finally, we sincerely thank the reviewers for their valuable comments, which have greatly contributed to improving the quality of the manuscript.

Sincerely yours,

Jihad Mallat, MD, PhD, FCCM

Gustavo A. Ospina-Tascon, MD, PhD
